# Evaluating temporal bone column density for optimized bone conduction implant placement

**DOI:** 10.3389/fsurg.2023.1293616

**Published:** 2023-11-30

**Authors:** Emile Talon, Franca Wagner, Stefan Weder, Lukas Anschuetz, Marco Caversaccio, Wilhelm Wimmer

**Affiliations:** ^1^ Department of Otorhinolaryngology, Head and Neck Surgery, Inselspital, Bern University Hospital, Bern, Switzerland; ^2^ ARTORG Center for Biomedical Engineering Research, University of Bern, Bern, Switzerland; ^3^ Department of Diagnostic and Interventional Neuroradiology, Inselspital, Bern University Hospital, Bern, Switzerland; ^4^ Department of Otorhinolaryngology, Klinikum rechts der Isar, Technical University of Munich, Munich, Germany

**Keywords:** bone column density, image-guided audiology, transmission efficiency, laser Doppler velocimetry, BAHA, quantitative computed tomography

## Abstract

**Introduction:**

An optimal placement of bone conduction implants can provide more efficient mechanical transmission to the cochlea if placed in regions with greater bone column density. The aim of this study was to test this hypothesis and to determine the clinical potential of preoperative bone column density assessment for optimal implant placement.

**Methods:**

Five complete cadaver heads were scanned with quantitative computed tomography imaging to create topographic maps of bone density based on the column density index (CODI). Laser Doppler vibrometry was used to measure cochlear promontory acceleration under bone conduction stimulation in different locations on the temporal bone, using a bone-anchored hearing aid transducer at frequencies ranging from 355 Hz to 10 kHz.

**Results:**

We found a statistically significant association between CODI levels and the accelerance of the cochlear promontory throughout the frequency spectrum, with an average increase of 0.6 dB per unit of CODI. The distance between the transducer and the cochlear promontory had no statistically significant effect on the overall spectrum.

**Discussion:**

We highlight the importance of bone column density in relation to the mechanical transmission efficiency of bone conduction implants. It may be worthwhile to consider column density in preoperative planning in clinical practice.

## 1. Introduction

Bone conduction implants are well established as a treatment for conductive and mixed hearing loss (1), as well as single-sided sensorineural deafness (2, 3). These implants operate by directly conveying vibrations to the inner ear through the bone conduction pathway. However, particularly in patients with mixed hearing loss, their use is restricted by the maximum output level and transmission efficiency, which limits the dynamic range available for acoustic stimulation (4, 5).

Several factors have an influence on the bone conduction pathway, such as the compression and expansion of the inner ear (6), the inertial properties of the cochlear fluids and ossicles (7, 8), intracranial transmission (9), and induction of sound pressure in the ear canal (10). In addition to these mechanisms, the attachment of the transducer to the temporal bone determines the efficiency of transmission to the cochlea (11, 12). Furthermore, the position of attachment to the temporal bone has been shown to influence transmission efficiency, with implants placed closer to the cochlea producing greater promontory motion during stimulation (13).

Preoperative planning was proposed to identify sites with enough bone thickness to allow screw fixation or implant placement (14, 15), especially where space is limited (e.g., in children) (16, 17). Although the assessment of temporal bone thickness has been well documented (18), the effect of the temporal bone mass distribution on the transmission of mechanical vibrations in bone conduction implants has yet to be explored in a clinical context. The temporal bone is a highly complex structure containing air cells and composed of different types of bone tissue, such as cortical bone, cancellous bone, and diploë, with varying densities (19). The transmission of energy from the transducer to the temporal bone should be influenced by the mechanical point impedance (20). Different bone column densities should therefore affect the local mechanical impedance as well as the impedance matching between the transducer and the skull. Our team has recently proposed the incorporation of bone column density, a measure of mass distribution in the transducer position, as a supplementary approach to identify areas that may be favorable for bone conduction implants (21). Bone density information can be retrieved indirectly during clinical practice, since preoperative computed tomography (CT) imaging is often performed routinely during candidacy checks (22, 23).

The purpose of this study was to determine the clinical potential of evaluating the distribution of temporal bone column density before surgery to facilitate optimized implant placement. We hypothesized that the placement of an implant in areas of higher column density could facilitate mechanical transmission of vibrations through the bone, ultimately resulting in more efficient transmission to the inner ear.

## 2. Materials and methods

### 2.1. Specimen preparation

To test the hypothesis, we conducted an *ex-vivo* study with five whole head cadaver specimens. The specimens were conserved using the Thiel method (24), which has been established as a viable model for experimental evaluation of the bone conduction pathway in anatomical specimens (25–27). Mastoidectomy and posterior tympanotomy were performed in nine out of ten ears to obtain a direct line of sight to the cochlear promontory, as required for laser Doppler velocimetry (LDV) measurements. The tenth temporal bone was not available for measurements due to a previous study that limited the integrity of the cochlea on this side. The research protocol was approved by our institutional review board (KEK-BE 2016-00887).

### 2.2. Temporal bone mass distribution

All specimens were imaged with a clinical CT scanner (Somatom Definition Edge, Siemens, Germany; 94 mA, 120 kV, voxel size: 0.15 × 0.15 × 0.2 mm^3^. The bone mineral density (expressed in mg_HA_ cm^−3^) of the tissue was estimated from the measured Hounsfield units, using a calibration phantom (QRM-BDC-6, QRM GmbH, Moehrendorf, Germany) as described by Talon et al. (21).

**Figure 1:**
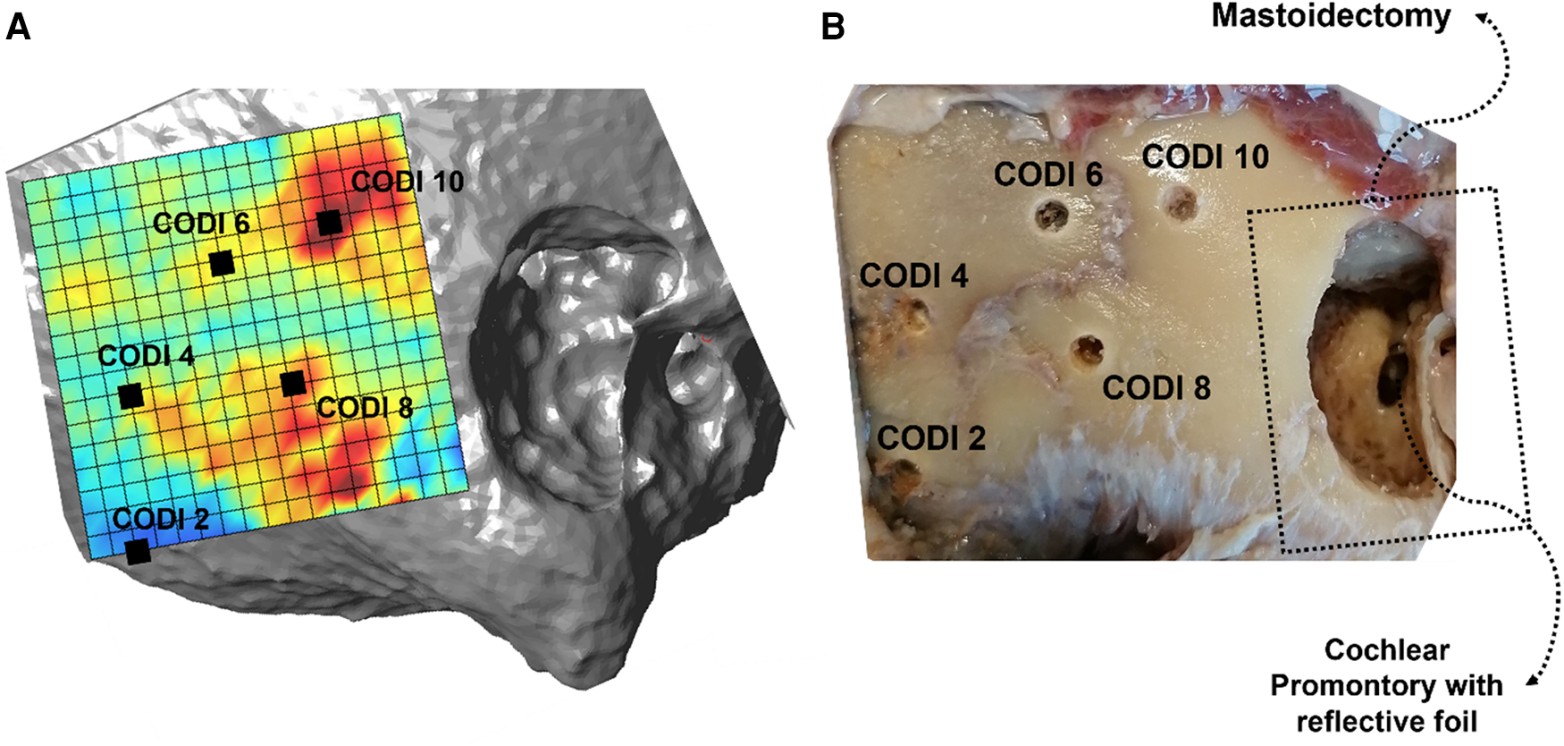
(**A**) Topographic column density index (CODI) map, with five selected implant positions covering 2–10 mg_HA_ mm^−2^. Regions with higher CODI levels indicate greater local temporal bone mass. (**B**) Corresponding situation with the holes drilled for transducer positioning in the specimen (“Head 18 right”).

To segment temporal bone and generate surface models, the open-source software 3D Slicer (28) was utilized. The surface models were then imported into a Matlab script (Mathworks Inc, Natick, MA, USA) to quantify the temporal bone mass distribution. For this purpose, we computed the bone column density index (CODI, expressed in mg_HA_ mm^−2^) within a retroauricular region of interest and visualized the densities as a topographic map (see Figure 1 left) (15, 21). The CODI specifies the accumulated bone mineral density along a probing trajectory over the full thickness of the temporal bone:

$$\mathrm{CODI}=\;\overset{N}{\underset{i=1}{\sum }}\rho_{i}\;\Delta d$$

where *N* denotes the total number of sampled voxels along the probing trajectory within the total temporal bone thickness, *ρ*_i_ is the bone mineral density (in mg_HA_ mm^−3^) of the voxel with index *i*, and Δ*d* is the sampling interval along the trajectory (in our case, 0.15 mm).

### 2.3. Transducer position and stimulation

For stimulation, the transducer of a bone-anchored hearing aid (BAHA 110 Power™, Cochlear, Australia) was driven by an external signal generator and audio analyzer (FX100, NTi Audio, Liechtenstein). Measurement traces were obtained by performing a sinusoidal sweep with an amplitude of 1V, spanning a frequency range from 100 Hz to 10 kHz and with 111 logarithmic frequency steps.

As with bone-anchored hearing aids, the transducer was attached to an abutment, which was fixed to a 3 mm titanium implant. The transducer positions were selected according to the topographic CODI maps to cover a variety of temporal bone mass distributions. A total of 40 transducer positions were selected among the specimens, with an average CODI of 7.8 mg_HA_ mm^−2 ^± 2.6 mg_HA_ mm^−2^ (range: 2–13 mg_HA_ mm^−2^) The positions of the transducers were transferred to the temporal bones using a self-built measuring device, referencing the upper posterior point on the edge of the mastoidectomy and the zygomatic line (15). At the positions indicated, holes of 3 mm diameter were drilled to a depth of 3 mm to insert the implant (see Figure 1 right). After drilling, CT scans were again performed using an identical imaging protocol and co-registered with the original CT images to determine the actual positions of the drilled holes and recalculate the corresponding CODI levels. In addition, the Euclidean distance from the transducer position to the center of the round window was computed using the 3D Slicer software.

### 2.4. Cochlear promontory acceleration

**Figure 2:**
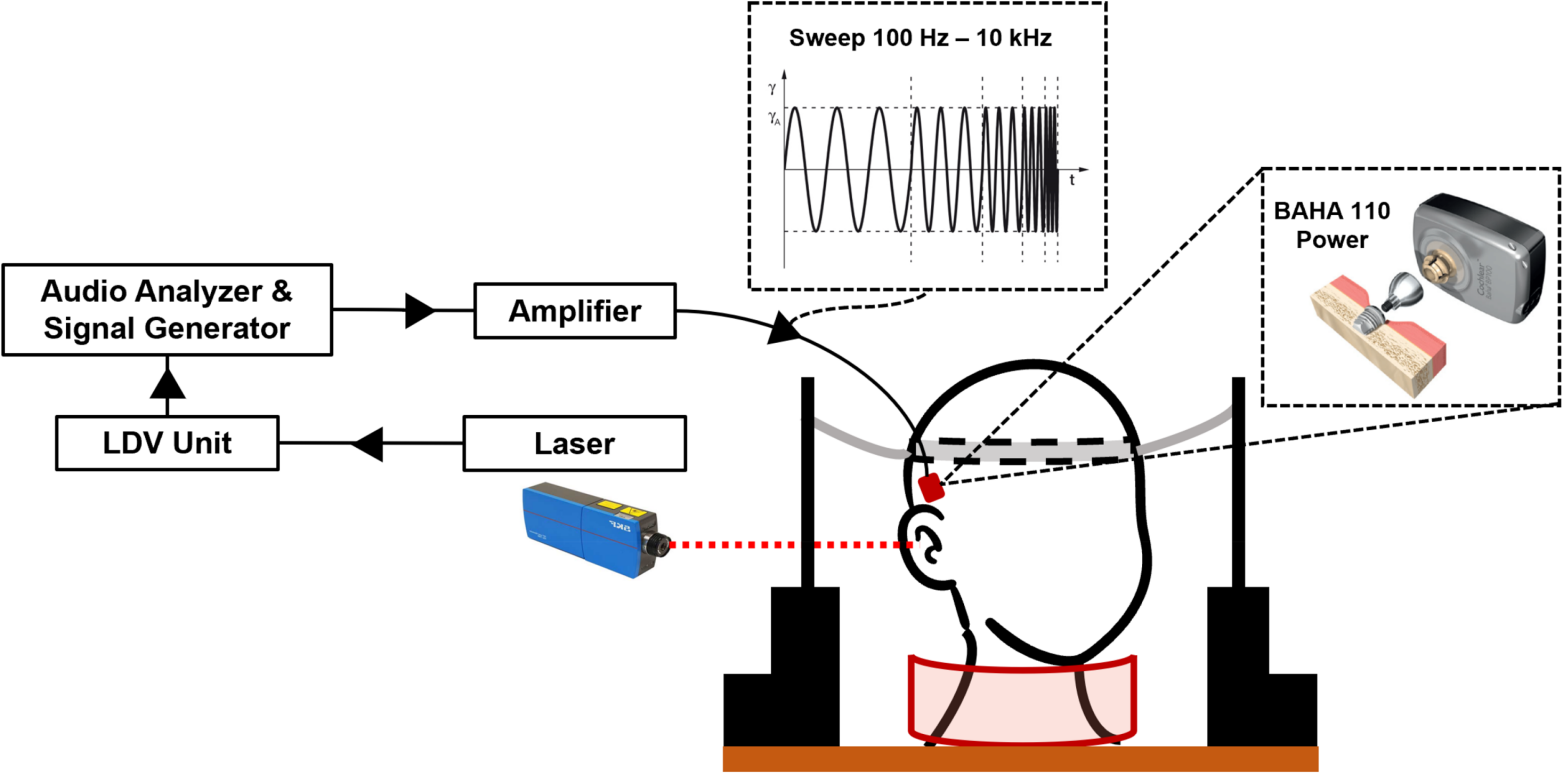
Overview of the experimental setup. The specimen is kept upright using a pillow placed around the neck and two lateral supports with an elastic headband (31). The audio analyzer generates the signal that is amplified to stimulate the transducer. The cochlear promontory velocity is measured using a laser Doppler vibrometer and converted to acceleration. After measurement, the transducer and implant are removed and placed in the next position indicated on the temporal bone.

An overview of the experimental setup is shown in Figure 2. We used a 1D LDV (VibroOne®, Polytec GmbH, Germany) to measure the velocity of the ipsilateral cochlear promontory during stimulation (29, 30). To enhance the measurement, a small piece of reflective foil with a surface area of 1 mm^2^ was carefully placed on the promontory (31). Measurements were made with the sensitivity set to 5 mm^s−1^ V^−1^ and only if a 40% signal strength or higher was achieved. At this signal strength or higher, the defined stability criterion of the analyzer for three consecutive measurements at a frequency being within 0.2 dB of each other could be met. The promontory acceleration was computed from the velocities. All measurements were carried out in a vibration-damped acoustic chamber.

### 2.5. Transducer output force level and accelerance

To normalize the promontory acceleration per unit force (referred to as accelerance), the output force level (OFL, expressed in dB re 1 µN) of the transducer was measured using a calibrated artificial mastoid (Type 4930, Brüel & Kjær, Denmark) (30, 32). On the artificial mastoid, the transducer was loaded with a static force of 5 N and driven with the same frequency sweep signal as for the experiments (see Supplementary Figure S1 in the Supplementary Information). The cochlear promontory accelerance is expressed in dB re 1 m s^−2^ N^−1^. For data analysis, frequencies between 350 and 10 kHz were considered, as lower frequencies were associated with unreliable measurements, mainly attributable to noise.

### 2.6. Statistical analysis

Statistical analysis on the promontory accelerance was performed with linear mixed-effects models. First, an overall model on the total power within octave bands with center frequencies at 0.5, 1, 2, 4 and 8 kHz) was estimated. CODI levels, the distance between the transducer position and the center of the round window, and the octave band were considered fixed effects. In general, a correlation between increasing distances from the cochlear promontory and lower CODI levels can be expected (21). However, analysis of the variance inflation factor indicated low collinearity among the predictor variables in the model. This is supported by the factors obtained for each variable: CODI (1.95), distance (1.95), and octave band (1.0). To understand whether the CODI was more relevant at specific octave bands, separate linear mixed-effects models for the different frequency bands were fit. A random intercept of ear side nested within specimens was included in all models to account for paired measurements (anatomical variation and differences in experimental conditions, e.g., alignment of LDV for each side). A significance level of 0.05 was used for all comparisons. Statistical analysis was performed using R Studio and the “lme4” package (33).

## 3. Results

### 3.1. Transducer position transfer from plan to specimen

The average positioning error caused by the transfer from the planned position to the actual drilled position in the samples was 3.2 mm (standard deviation, SD = 1.8 mm). This change in positions resulted in an average CODI difference of 1.3 mg_HA_ mm^−2^ (SD = 1.4 mg_HA_ mm^−2^).

### 3.2. Cochlear promontory accelerance

**Figure 3:**
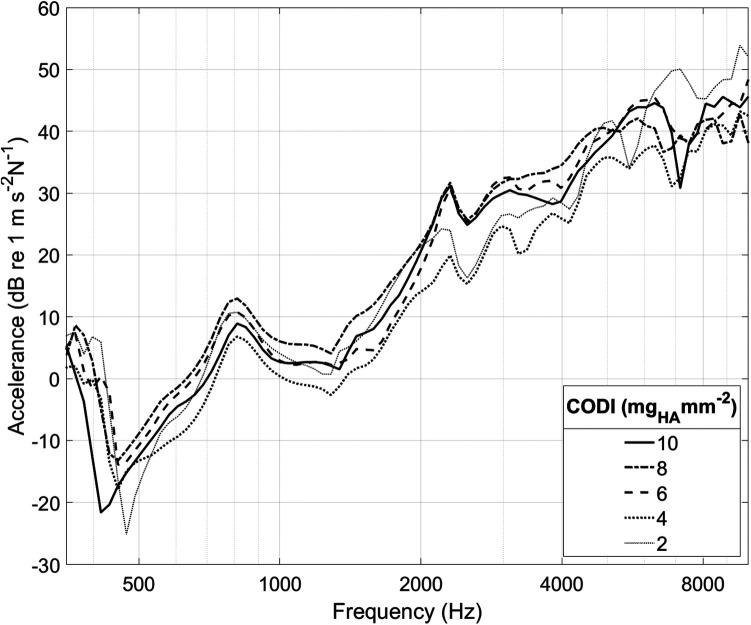
Cochlear promontory accelerance plotted over frequency for specimen “head 18”. Each curve corresponds to a different transducer position with a specific column density index (CODI; in mg_HA_ mm^−2^; see Figure 1).

Figure 3 illustrates the accelerance over frequency for different levels of CODI in a specimen. In all measurements, a pronounced antiresonance was present up to 700 Hz. The frequency of this antiresonance is believed to depend on the distance between the implantation site and the external ear canal (13). No such association was apparent in our data (see Supplementary Figure S3 in the Supplementary Information).

### 3.3. Bone column density and promontory accelerance

**Figure 4:**
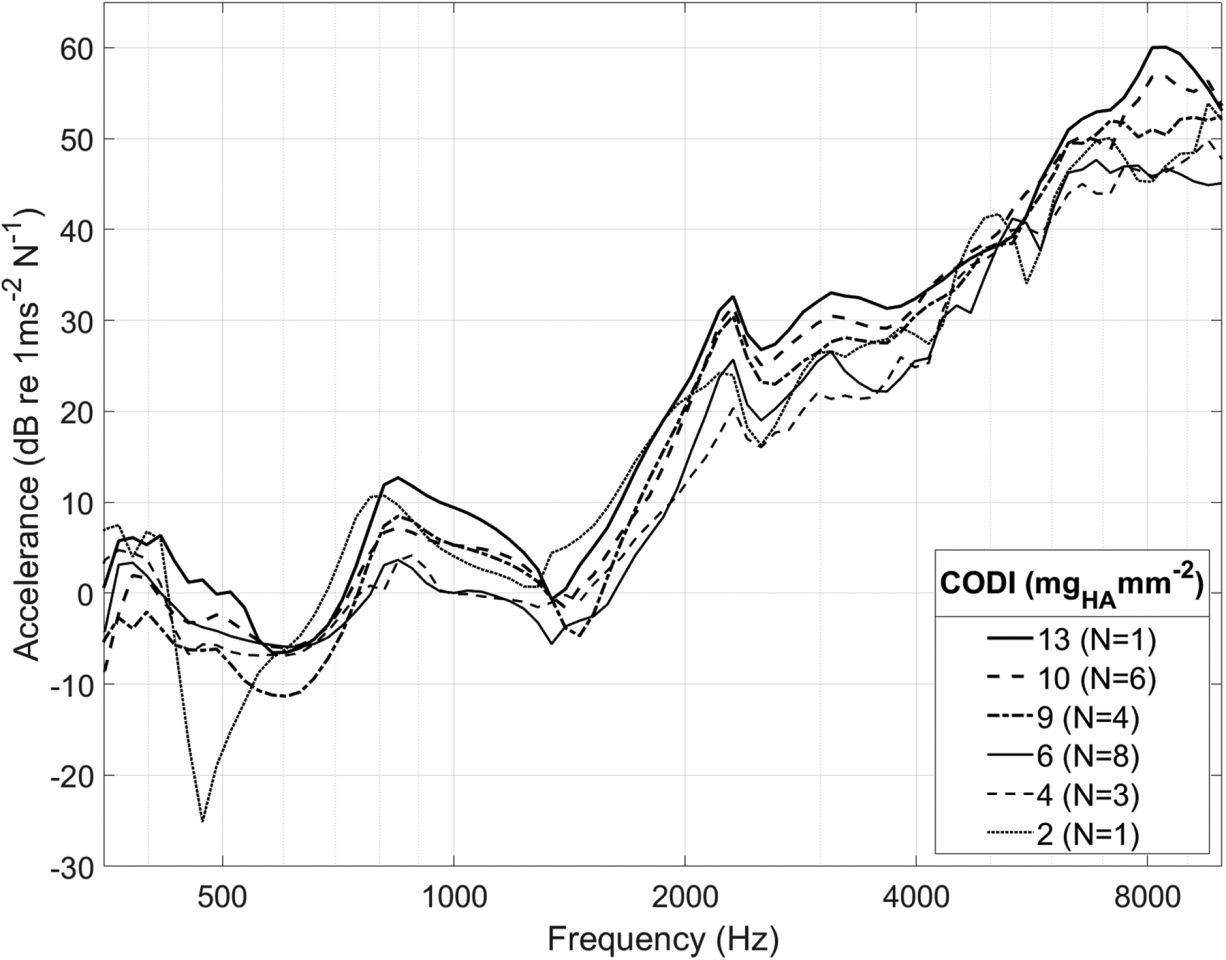
Spectrum of cochlear promontory accelerance averaged among all ear sides. For clarity, only selected CODI levels (in mgHA mm^−2^) are shown; *N*, number of samples.

Figure 4 shows the promontory accelerance measured for different CODI levels averaged for all specimens. Our results are consistent with data reported in the literature (13, 25, 30, 36, 37) (see Supplementary Figure S4 in the Supplementary Information).

**Figure 5:**
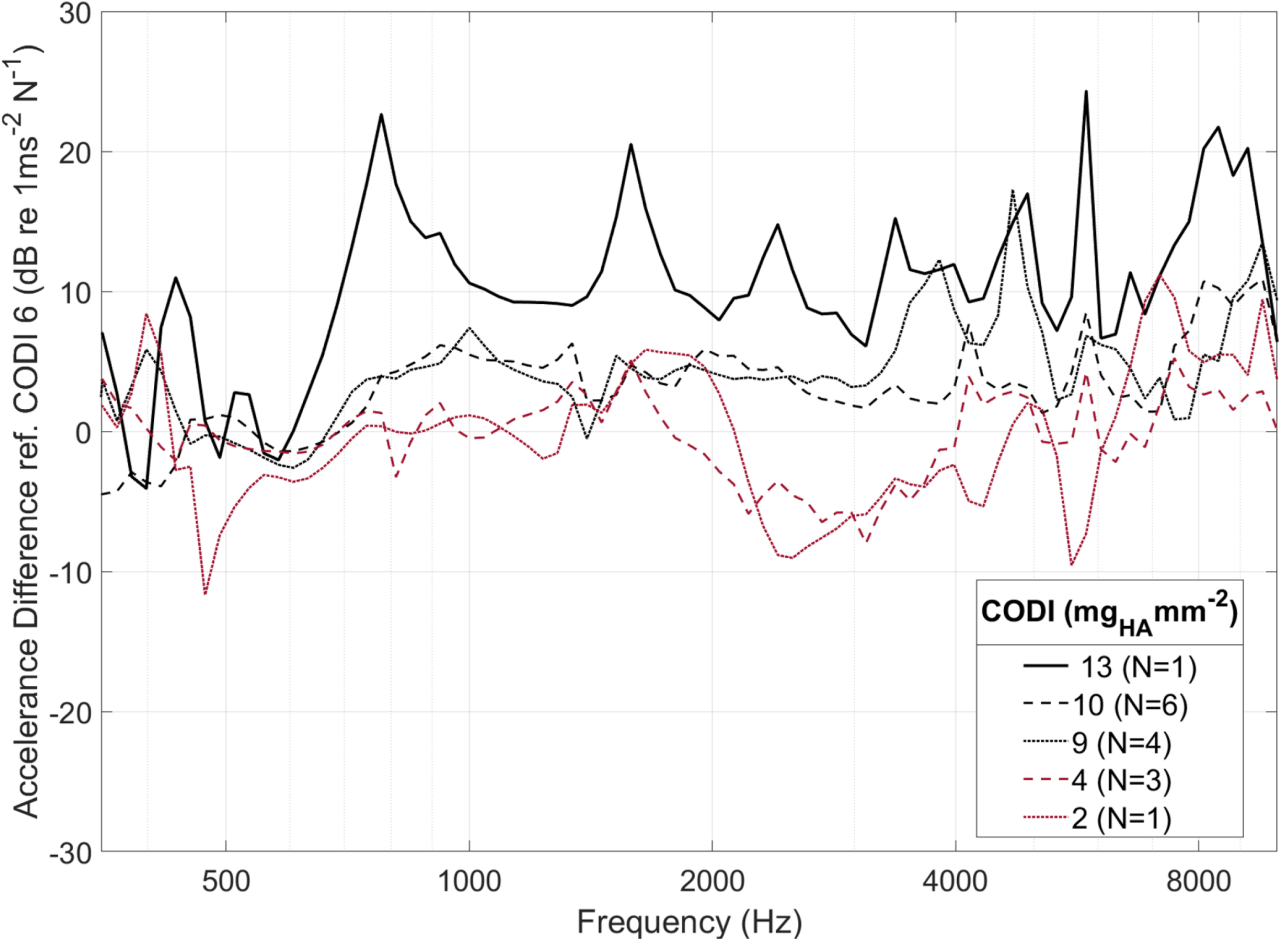
Averaged within-specimen accelerance differences for varying column density indices (CODI) with respect to a reference CODI of 6 mg_HA_ mm^−2^. For clarity, only selected CODI levels are shown; *N*, number of samples.

As individuals have distinct temporal bone morphology, variation between subjects is expected (37). For example, at transducer positions with a CODI level of 6 mg_HA_ mm^−2^, the promontory accelerance measured showed a standard deviation of 4.5 dB throughout the frequency spectrum for all specimens. Figure 5 shows the differences with respect to CODI levels of 6 mg_HA_ mm^−2^ averaged for all ears. A notable association is evident between regions characterized by increased column densities and elevated promontory accelerance. On average, the difference in accelerance between transducers placed at the highest and lowest CODI levels (13 and 2 mg_HA_ mm^−2^, respectively) was 20 dB, reaching peaks greater than 40 dB around 6 kHz. This is confirmed by the statistically significant effect of CODI levels on promontory accelerance (*p* < 0.001) in our data. An increment of 1 mg_HA_ mm^−2^ is associated with an average increase in accelerance of 0.6 dB (see Table 1). In the individual octave band linear mixed-effects models, a statistically significant association between CODI and the accelerance was found only at 1, 2, and 8 kHz. Each one-unit increase in CODI corresponded to accelerance amplifications of 0.1 dB (not significant), 0.7 dB (*p* = .007), 0.8 dB (*p* = .002), 0.1 dB (not significant), and 0.8 dB (*p* = .03) for the 0.5, 1, 2, 4, and 8 kHz octave bands, respectively (Supplementary Tables S1–S5 in the Supplementary Information).

**Table 1:** Linear mixed-effects model summary for cochlear promontory accelerance (in dB re 1 m s*^−^*^2^ N*^−^*^1^) across all frequencies.

	Estimate	Std. Err.	*p*-value
Intercept	11.90	4.10	.**005**
Column density index (CODI; in mg_HA_ mm*^−^*^2^)	0.58	0.14	*<*.**001**
Distance to promontory (in mm)	−0.10	0.07	.14
Band 1 kHz	4.01	0.82	*<*.**001**
Band 2 kHz	22.65	0.82	*<*.**001**
Band 4 kHz	36.82	0.82	*<*.**001**
Band 8 kHz	51.11	0.82	*<*.**001**

Bold values indicate statistically significant effects.

### 3.4. Transducer distance and promontory accelerance

An average distance of 47 mm between the transducer position and the round window was measured, with a range spanning from 38 mm to 61 mm. Previous studies suggested a correlation between the transducer–cochlea distance and the efficacy of bone conduction implants (13). Over the whole frequency spectrum, our data show no statistically significant association between distances and cochlear promontory accelerance; see Table 1).

In the models fit to the individual octave bands, we found a statistically significant, but weak, association between the transducer distance and the accelerance in the 4 kHz octave band only (Supplementary Tables S1–S5 in the Supplementary Information). A 1 mm increase in distance corresponds to a 0.23 dB decrease in accelerance (*p* = .04).

## 4. Discussion

We investigated the association between the distribution of temporal bone column density and the efficiency of bone conduction implants in transmitting mechanical vibrations to the inner ear. Our results demonstrate a significant association between higher bone column density and promontory accelerance, indicating improved transmission of vibrations. This finding supports our hypothesis that optimizing implant placement toward areas of higher column density could enhance mechanical transmission through the bone and improve the efficiency of bone conduction implants.

### 4.1. Bone column density and promontory accelerance

Based on our linearized analysis over the whole measured frequency spectrum, it is reasonable to anticipate a difference in acceleration levels of approximately 6 dB between locations with low (CODI 2) and high (CODI 12) bone column densities. The cochlear promontory acceleration is considered a plausible indicator for bone conduction hearing thresholds (6, 13, 37), although a direct quantitative association has not yet been established. We expect the variations of cochlear promontory accelerance levels to be reflected in audiological outcomes as variations aided hearing thresholds, however, prospective controlled studies are required to test this hypothesis.

For a frequency band-specific interpretation of our results, it can be helpful to consider the categorization of mechanical point impedance (20, 38). The skull impedance exhibits an antiresonance at around 150 Hz (20, 37). Below this antiresonance, the mechanical point impedance is predominantly governed by the mass of the skull. Above the antiresonance, but still in the low-frequency domain, the impedance is determined by the stiffness of the whole skull. This is reflected in our data, as locally estimated bone column density has no effect on the mechanical impedance in the 500 Hz octave band.

With increasing frequencies, the mechanical point impedance is increasingly determined by the local bone properties around the implant. A higher column density at the implant location (as expressed by the CODI) results in an interface with greater stiffness, and therefore an increased mechanical point impedance. A greater stiffness in the bone results in better transmission of vibrations, as there is less energy loss due to deformation and damping. This is evident in our results, as the influence of column density on promontory accelerance is statistically significant in the 1 kHz and 2 kHz octave bands. Interestingly, we did not observe this behavior in the 4 kHz octave band. In this region, the system is generally transitioning from being fully regulated by the stiffness of the interface to a system regulated by the bone mass surrounding the implantation site (20). An antiresonance in the mechanical point impedance around 4 kHz was found, depending on the device, fixation method and implant positioning used in computational studies (39), together with a shift in phase from negative to positive values, in both computational and experimental studies (20, 39). At frequencies above 6 kHz, the system is fully controlled by the mass of the interface region (20). The CODI, being linked to the mass of the bone surrounding the implantation site, has a strong impact on the transmission levels in the high-frequency region, as highlighted by our results. At these frequencies (1–2–8 kHz) this would mean that between the coupling and the skull the mismatch is smaller.

### 4.2. Transducer distance and promontory accelerance

The spatial relationship between the implant and the cochlea has significant implications for transmission efficiency, prompting ongoing research on the extent of these effects. Previous investigations have illustrated that bone conduction transmission is improved when the transducer is positioned closer to the cochlea (13, 37, 40). Our study identified a distinct impact of distance, specifically within the 4 kHz frequency band, where we observed a reduction in transmission of 2 dB per centimeter. This observation aligns with the findings of Stenfelt and Goode (37), who reported a decrease of 1.5 dB per centimeter. It not yet known whether the overall effect of distance on transmission efficiency is primarily attributable to the actual distance or to the bone density, which tends to decrease with longer distances. In addition, future investigations should encompass the computation of the shortest bone-borne distance to the cochlea. Moreover, the presence of the squamosal suture along the transmission path is believed to be a significant factor in reducing vibration intensity, resulting in differences of up to 4 dB at higher frequencies (13).

### 4.3. Comparison with other studies

The LDV measurement setup, which is a commonly used objective measurement technique, was employed to evaluate the potential effectiveness of hearing rehabilitation (30). Our results are in agreement with measurements obtained in other studies with 1D LDV (13, 29, 36) and studies with 3D LDV measurements (34, 37) (see Supplementary Figure S2 in the Supplementary Information). Other conditions strongly influence the output of the low-frequency domain, such as the fact that the head is separated from the rest of the body (30) and the method used for fixation of the neck (31). A pronounced antiresonance is found in all measurements in the 0.5–0.8 kHz domain, and it is strongly linked to the interaction between the mass and the compliance of the skull and the compliance mismatch of the interface (13, 20, 37, 41), together with the exact location of the implantation site (13). All the small resonances and antiresonances in the higher frequency range had a very high inter-subject variability, suggesting that they are highly dependent on the head morphology, dimensions, and bone characteristics (20).

### 4.4. Potential clinical application

The correlation between bone density and orthopedic device stability has been well-established in the literature (42–45). In the field of otology, researchers have quantified bone mineral density in otosclerosis patients (46) and explored the relationship between density and age (21, 47). However, the impact of bone density in the temporal bone on the transmission of mechanical vibrations remains relatively underexplored in clinical research. Our approach holds promise for aiding surgeons in preoperative evaluations to determine optimal implant positions, akin to assessing temporal bone thickness using CT data (15, 48, 49). We expect that the CODI values determined in living subjects are comparable to the range of CODI values reported here, as previous studies have shown a negligible influence of the Thiel fixation method on bone mineral density (21, 50). An extended implantation index could be developed for clinical use that takes into account column density and geometrical information to guide better surgical decision-making. The accuracy with which the planned position can be transferred the to the patient is a key factor in clinical applicability. In this study we used a custom-made measuring tool to reduce misplacement. Image-guided navigation techniques could also be employed, but the effort required for their preparation would need to be justified.

### 4.5. Study limitations

Our study has limitations that need to be considered. The use of *ex-vivo* models may not fully replicate *in vivo* conditions, particularly regarding osseointegration effects. Nevertheless, the use of cadaver specimens allowed for controlled experiments and precise measurements. Certain factors relevant to bone conduction sound transmission, such as skull condition and sample manipulation, were not considered in our study (40). For instance, the gray matter volume and consistency in cadaver samples significantly differ from those in living humans. Nonetheless, we found that skull vibrations remained decoupled from the underlying gray matter above 100 Hz (37). Quantifying the influence of these parameters on vibration transmission is challenging.

Each sample underwent mastoidectomy to access the cochlear promontory for LDV measurements. The larger access enhanced laser signal detection and resulted in quicker and more reliable readings. However, the absence of mastoid bone may have altered the vibration patterns, potentially influencing the impact of the bone conduction implant (CODI) on mechanical vibration transmission. Although *in vivo* measurements have not shown substantial alterations in temporal bone compliance or skull impedance due to mastoidectomy (20), it is nevertheless recommended to explore alternative cochlear promontory access methods, such as a tympanomeatal flap.

For bone mineral density calibration in CT images, we used a single scan on a phantom, as previously described by Talon et al. (21). This calibration established a linear relationship between Hounsfield units and bone mineral density. However, it is important to note that this relationship might change over time, necessitating recalibration before each CT scan. Various calibration methods, as discussed by Goodsitt et al. (51), could slightly influence the scaling factor and consequently the CODI values measured. In selecting different transducer positions, we ensured that they were located in areas with constant CODI values within an area of at least 4 mm^2^, thus guaranteeing that the entire surrounding bone region in contact with the abutment exhibited the same CODI level. However, if the implant is placed in a highly restricted region with a constant CODI value, the validity of the CODI index may be limited.

## 5. Conclusions

Our study reveals a significant association between the distribution of temporal bone column density and the efficiency of bone conduction implants in transmitting mechanical vibrations to the inner ear. Higher bone column density was correlated with improved promontory accelerance, indicating enhanced transmission of vibrations. This finding supports the potential clinical usefulness of evaluating bone column density before surgery to facilitate optimized implant placement. Preoperative planning based on bone density information could lead to improved surgical decision-making and potentially enhanced audiological outcomes for patients undergoing bone conduction implantation. However, *in vivo* studies with larger sample sizes are required to confirm these findings and explore the practical clinical implications of incorporating bone density information.

## Data Availability

The original contributions presented in the study are included in the article/**Supplementary Material**, further inquiries can be directed to the corresponding author.
