# Supplementary material for: Evaluating temporal bone column density for optimized bone conduction implant placement
Source: Front Surg. 2023 Nov 30;10:1293616. doi: 10.3389/fsurg.2023.1293616 (PMC10720247; doi:10.3389/fsurg.2023.1293616)
Supplement: Supplementary file 1 [file Datasheet1.pdf]

Supplementary Information

Supplemental Figures 2

Supplemental Tables 3

Supplemental Figures

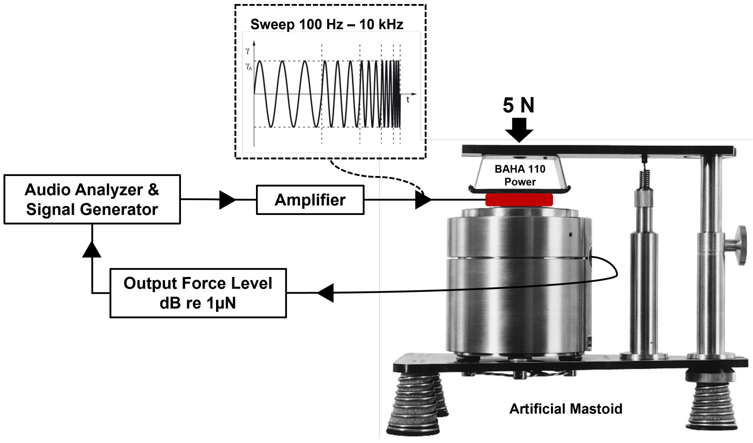

Figure S.1: The set-up used to compute the output force level (OFL) of the transducer used. The transducer from the BAHA is externally driven and stimulated with a sweep over the frequency spectrum. An artificial mastoid (Type 4930, Brüel & Kjær, Denmark) is used to convert the force generated by the electromagnetic transducer in the BAHA into an electrical signal, then analyzed in the audio analyzer.

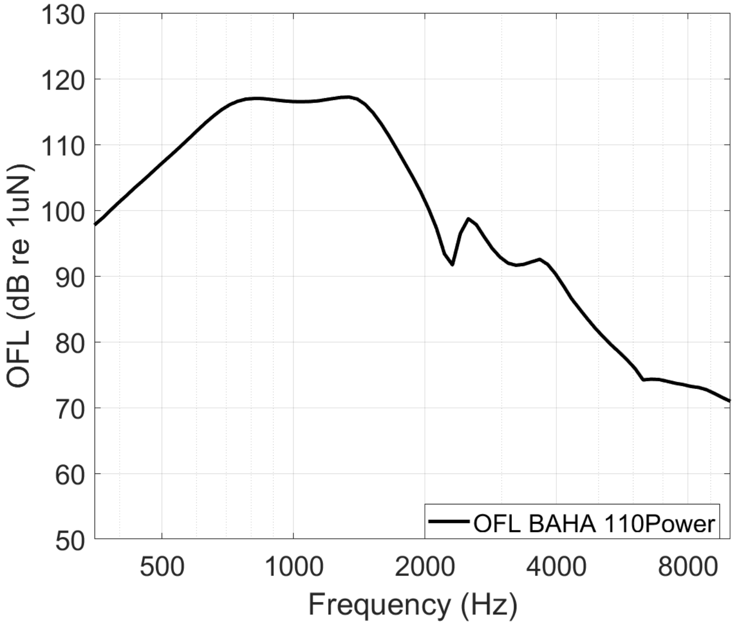

Figure S.2: Output force level of the transducer used in the experiments. The force generated varies from 60 to 120 dB re 1 µN and has its peak around 1 kHz.

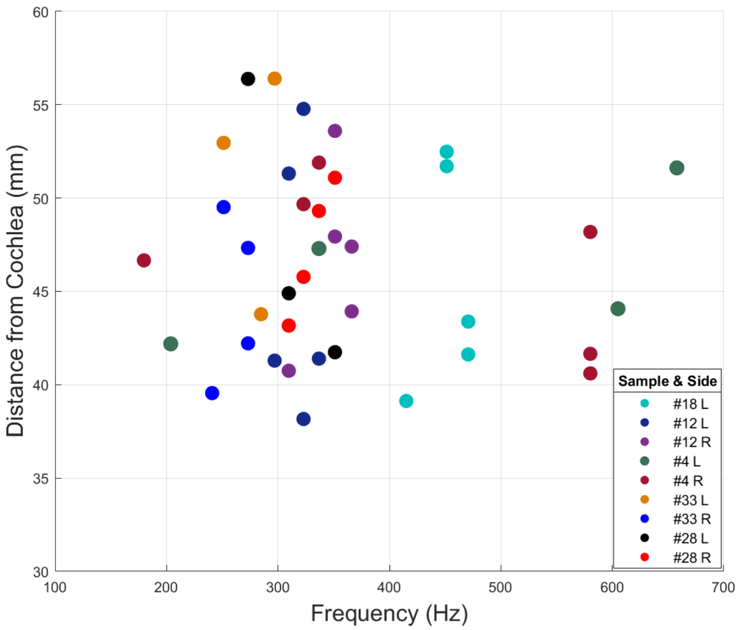

Figure S.3: Scatter plot of the antiresonance of each measurement in relation to the distance from the implantation site to the cochlea. No statistically significant correlation was found.

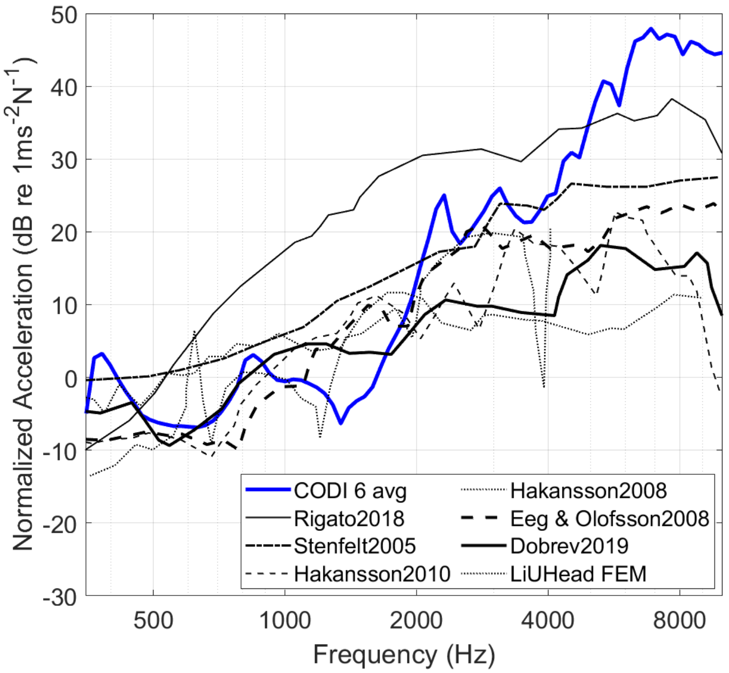

Figure S.4: The average of all the measurements obtained in the bone regions characterized by CODI of 6 (in blue) compared to the results obtained in other experimental studies [6, 7, 3, 4, 2, 1] or in finite element model simulations [5]. The results presented in this study are consistent with literature.

Supplemental Tables

Table S.1: Linear mixed effect model results comparing the effect of column density index (CODI) and the distance from the stimulator to the cochlear promontory (CP) on the levels of CP acceleration in the 0.5 kHz frequency band. The CODI and the distance to the CP are expressed as continuous independent variables.

	**Estimate**	**Std. Err.**	***p-value***
**Intercept**	8.10	6.11	.20
**CODI**	0.05	0.21	.81
**Distance to CP**	0.07	0.10	.51

Table S.2: Linear mixed effect model results comparing the effect of column density index (CODI) and the distance from the stimulator to the cochlear promontory (CP) on the levels of CP acceleration in the 1 kHz frequency band. The CODI and the distance to the CP are expressed as continuous independent variables.

	**Estimate**	**Std. Err.**	***p-value***
**Intercept**	15.50	6.93	$.03^{*}$
**CODI**	0.70	0.24	$.007^{**}$
**Distance to CP**	-0.11	0.12	.33

Table S.3: Linear mixed effect model results comparing the effect of column density index (CODI) and the distance from the stimulator to the cochlear promontory (CP) on the levels of CP acceleration in the 2 kHz frequency band. The CODI and the distance to the CP are expressed as continuous independent variables.

	**Estimate**	**Std. Err.**	***p-value***
**Intercept**	35.93	6.86	$< .001^{***}$
**CODI**	0.80	0.24	$.002^{**}$
**Distance to CP**	-0.16	0.11	0.16

Table S.4: Linear mixed effect model results comparing the effect of column density index (CODI) and the distance from the stimulator to the cochlear promontory (CP) on the levels of CP acceleration in the 4 kHz frequency band. The CODI and the distance to the CP are expressed as continuous independent variables.

	**Estimate**	**Std. Err.**	***p-value***
**Intercept**	58.08	6.37	$< .001^{***}$
**CODI**	0.13	0.22	0.58
**Distance to CP**	-0.23	0.11	0.04^{*}

Table S.5: Linear mixed effect model results comparing the effect of column density index (CODI) and the distance from the stimulator to the cochlear promontory (CP) on the levels of CP acceleration in the 8 kHz frequency band. The CODI and the distance to the CP are expressed as continuous independent variables.

	**Estimate**	**Std. Err.**	***p-value***
**Intercept**	69.75	9.60	$< .001^{***}$
**CODI**	0.75	0.33	0.03^{*}
**Distance to CP**	-0.27	0.16	0.09

References

[1] Ivo Dobrev et al. “Experimental investigation of promontory motion and intracranial pressure following bone conduction: Stimulation site and coupling type dependence”. In: *Hearing Research* 378 (July 2019), pp. 108–125. ISSN: 0378-5955. DOI: 10.1016/J.HEARES.2019.03.005.

[2] Måns Eeg-Olofsson et al. "Transmission of bone-conducted sound in the human skull measured by cochlear vibrations". In: *http://dx.doi.org/10.1080/14992020802311216* 47.12 (Dec. 2009), pp. 761–769. DOI: 10.1080/14992020802311216. URL: https://www.tandfonline.com/doi/abs/10.1080/14992020802311216.

[3] Bo Håkansson et al. "A novel bone conduction implant (BCI): Engineering aspects and pre-clinical studies". In: *International Journal of Audiology* (2010). ISSN: 1708-8186. DOI: 10.3109/14992020903264462. URL: https://www.tandfonline.com/action/journalInformation?journalCode=iija20.

[4] Bo Håkansson et al. "Percutaneous Versus Transcutaneous Bone Conduction Implant System: A Feasibility Study on a Cadaver Head". In: *Otology and Neurotology* 29.8 (2008), pp. 1132–1139. ISSN: 15317129. DOI: 10.1097/MAO.0b013e31816fdc90.

[5] Srdjan Prodanovic and Stefan Stenfelt. "Review of Whole Head Experimental Cochlear Promontory Vibration with Bone Conduction Stimulation and Investigation of Experimental Setup Effects:" in: *https://doi.org/10.1177/23312165211052764* 25 (Oct. 2021). ISSN: 23312165. DOI: 10 . 1177 / 23312165211052764. URL: https : / / journals . sagepub . com / doi / full / 10 . 1177 / 23312165211052764.

[6] Cristina Rigato et al. "Direct bone conduction stimulation: Ipsilateral effect of different transducer attachments in active transcutaneous devices". In: *Hearing Research* 361 (Apr. 2018), pp. 103–112. ISSN: 0378-5955. DOI: 10.1016/J.HEARES.2018.01.007.

[7] Stefan Stenfelt and Richard L. Goode. "Transmission properties of bone conducted sound: Measurements in cadaver heads". In: *The Journal of the Acoustical Society of America* 118.4 (2005), pp. 2373–2391. ISSN: 0001-4966. DOI: 10.1121/1.2005847.
